# Insulin Secretion Defect in Children and Adolescents with Obesity: Clinical and Molecular Genetic Characterization

**DOI:** 10.1155/2024/5558634

**Published:** 2024-03-20

**Authors:** Helena Enders-Seidlitz, Klemens Raile, Maolian Gong, Angela Galler, Peter Kuehnen, Susanna Wiegand

**Affiliations:** ^1^Charité Universitätsmedizin Berlin, Berlin, Germany; ^2^Vivantes Klinikum, Berlin, Germany; ^3^Max Delbrück Center for Molecular Medicine, Berlin, Germany

## Abstract

**Introduction:**

Childhood obesity is increasing worldwide and presents as a global health issue due to multiple metabolic comorbidities. About 1% of adolescents with obesity develop type 2 diabetes (T2D); however, little is known about the genetic and pathophysiological background at young age. The objective of this study was to assess the prevalence of impaired glucose regulation (IGR) in a large cohort of children and adolescents with obesity and to characterize insulin sensitivity and insulin secretion. We also wanted to investigate adolescents with insulin secretion disorder more closely and analyze possible candidate genes of diabetes in a subcohort.

**Methods:**

We included children and adolescents with obesity who completed an oral glucose tolerance test (OGTT, glucose + insulin) in the outpatient clinic. We calculated Matsuda index, the area under the curve (AUC (Ins/Glu)), and an oral disposition index (ISSI-2) to estimate insulin resistance and beta-cell function. We identified patients with IGR and low insulin secretion (maximum insulin during OGTT < 200 mU/l) and tested a subgroup using next generation sequencing to identify possible mutations in 103 candidate genes.

**Results:**

The total group consisted of 903 children and adolescents with obesity. 4.5% showed impaired fasting glucose, 9.4% impaired glucose tolerance, and 1.2% T2D. Matsuda index and Total AUC (Ins/Glu) showed a hyperbolic relationship. Out of 39 patients with low insulin secretion, we performed genetic testing on 12 patients. We found five monogenetic defects (ABCC8 (*n* = 3), GCK (*n* = 1), and GLI2/PTF1A (*n* = 1)).

**Conclusion:**

Using surrogate parameters of beta-cell function and insulin resistance can help identify patients with insulin secretion disorder. A prevalence of 40% mutations of known diabetes genes in the subgroup with low insulin secretion suggests that at least 1.7% of patients with adolescent obesity have monogenic diabetes. A successful molecular genetic diagnosis can help to improve individual therapy.

## 1. Introduction

Obesity is one of the most common chronic diseases in childhood and adolescence and has become a significant challenge to our healthcare system over recent decades [1, 2]. The onset of the COVID-19 pandemic has further amplified this challenge, notably accelerating the increase in BMI [3]. Obesity is commonly associated with secondary diseases in the context of metabolic syndrome, encompassing hypertension, dyslipidemia, and impaired glucose regulation [4].

The term “diabetes mellitus” describes a complex metabolic disorder characterized by chronic hyperglycemia attributed to defects in insulin secretion and/or insulin action, comprising various diabetes types with distinct pathogenesis and clinical features [5]. Besides type 1 diabetes, which accounts for more than 90% of pediatric cases in Western countries [6], T2D is becoming increasingly important in at-risk populations due to the mounting prevalence of obesity [7, 8]. Monogenic forms of diabetes account for approximately 1-4% of all diabetes cases in childhood and adolescence. The term “Maturity Onset Diabetes of the Young” (MODY) describes a group of monogenic forms of diabetes that occur as dominantly inherited, nonautoimmune diabetes in childhood, adolescence, or young adulthood (usually <25 years of age) [9]. 14 MODY forms are distinguished, which are due to (functionally relevant) mutations in various genes that all play an elemental role in pancreatic beta-cell development or function [10].

For a small subset of children and adolescents, the classification of diabetes mellitus is a diagnostic challenge that has extensive consequences regarding the therapy. Despite clinical indicators such as obesity or a positive family history pointing toward specific diabetes types, some forms of diabetes can present very heterogeneously [11]. In addition to the assessment of diabetes-associated autoantibodies, the performance of an OGTT, and the measurement of HbA1c and C-peptide levels, molecular genetic testing emerges as a valuable tool in differential diagnostics [11]. Indices calculated from glucose and insulin values fasting and during the OGTT are surrogate parameters for estimating insulin resistance and beta-cell function since the hyperinsulinemic-euglycemic clamp technique is not feasible in clinical routine [12].

The dynamic interplay between insulin demand and secretion is crucial in maintaining normal glucose homeostasis, with insulin resistance and beta-cell function playing pivotal roles in the development of T2D in childhood and adolescence [13]. The interaction between insulin sensitivity and insulin secretion can be described as a hyperbolic relationship observed under normal glucose tolerance [14]. Decreased insulin sensitivity can be compensated for by increased insulin secretion, keeping the product of these parameters, also known as the disposition index, unchanged [15]. This index is a much more accurate reflection of beta-cell function in the presence of prevailing insulin resistance than assessing insulin secretion alone [16].

Considering the development of diabetes, it is challenging to distinguish disturbed beta-cell function from prevailing insulin resistance since both factors contribute to its manifestation. The lack of compensation for decreased insulin sensitivity by an increased insulin secretion leads to a loss of the hyperbolic correlation and a decrease in the disposition index. Diabetes manifestation can be described as a “two-hit” disease, where early insulin inevitably involves disturbed insulin secretion, leading subsequently to hyperglycemia and the onset of type 2 diabetes [17].

Genetic factors play a significant role, particularly concerning beta-cell function. Children and adolescents with MODY or T2D often present a positive family history of diabetes [18]. Interestingly, numerous candidate genes associated with polygenic and multifactorial T2D are colocated with genes implicated in monogenic diabetes, hinting at shared signaling and metabolic pathways affecting both conditions [19, 20].

Successful molecular genetic diagnosis of monogenic diabetes is essential due to its profound implications on clinical course and therapeutic consequences. A successful diagnosis ideally prompts a pathogenesis-oriented change in therapy, enhances the predictability of disease progression, and enables a quantified assessment of risk among relatives [10].

Our objective was to assess the prevalence of impaired glucose regulation and T2D in a large cohort of obese children and adolescents and to characterize insulin sensitivity and secretion using OGTT data. Furthermore, we investigated a pilot cohort with evidence of insulin secretion disorder and screened for rare, pathogenic mutations of insulin secretion (monogenetic forms of diabetes, MODY).

## 2. Materials and Methods

### 2.1. Recruitment and Study Participants

Our study involved 903 children and adolescents regularly attending the obesity outpatient clinic between January 2010 and March 2015. All individuals were aged between 2 and 19 years, with a BMI above the 90^th^ percentile. Exclusion criteria were syndromic obesity, secondary obesity, other endocrine diseases, and type 1 diabetes, according to ISPAD guidelines. Each participant underwent an OGTT with the measurement of glucose and insulin levels. Prior to the investigation, participants adhered to a high-carbohydrate diet (>150 g/day) for three days and maintained a fasting period of at least 10 hours. Oral glucose loading was 1.75 g glucose/kg body weight up to a maximum of 75 g glucose. Plasma glucose and serum insulin levels were collected at intervals of 0, 30, 60, 90, and 120 minutes. The patients maintained physical rest throughout the test. Interpretation of the OGTT results, as well as the definition and classification of T2D, IGT, and IFG, followed ADA guidelines [21]. We routinely recorded a general pediatric status, including body length, weight, and blood pressure measurements. BMI was calculated and assessed according to age- and sex-specific percentile curves from Kromeyer-Hauschild (German reference data) [22]. Clinical parameters of patients diagnosed with IGR were collected, encompassing age at diagnosis, treatment modality, metabolic control (HbA1c) during follow-up, and family medical history.

### 2.2. Calculations

Several indices were calculated as surrogate parameters of insulin resistance and beta-cell function (see supplementary materials (available here)). The Homeostatic Model Assessment of Insulin Resistance (HOMA-IR) was computed from fasting insulin and glucose values and served as a marker for hepatic insulin resistance [23]. Age-specific reference values are available from Allard et al. [24]. The Matsuda index (WBISI) was calculated as a surrogate parameter for peripheral insulin sensitivity [25]. The total area under the curve (AUC (Ins/Glu)) indicates beta-cell function and was computed using the trapezoidal rule [26]. Additionally, the Insulin Secretion-Sensitivity Index-2 (ISSI-2), an oral disposition index, was calculated from the product of the Matsuda index and Total AUC (Ins/Glu) [27].

We defined suspected insulin secretion disorder as follows: criteria of IGT or T2D were fulfilled, and insulin levels did not rise above 200 mU/l during OGTT.

### 2.3. Genetic Testing

We obtained approval from the Charité ethics committee (EA2/062/15), and all patients and their caregivers gave informed consent. In a pilot study, out of 39 children and adolescents with IGR and suspected insulin secretion disorder, we tested 12 patients for candidate genes by next generation sequencing (NGS). For this project, a custom-designed assay was designed by Illumina. It contained the following genes or gene loci:

- 38 genes of monogenic forms of diabetes and diabetes syndromes
- 44 genes linked to T2D susceptibility loci
- 17 genes derived from experimental studies
- 3 genes associated with congenital hyperinsulinism
- 1 pancreatic aplasia locus (for detailed information about all genes, see supplementary materials)

A total of 1287 target regions were covered. Sequencing procedures were conducted utilizing an Illumina HiSeq2000 (Illumina, San Diego). Bioinformatic sequencing data analysis was performed by mapping sequences according to the human reference genome (Hg19).

### 2.4. Statistical Analysis

We used the program “IBM SPSS Statistics,” version 22 (IBM, Armonk, New York) for statistical analysis. Descriptive statistics for the patient cohort were derived using frequencies and cross tables. Measures of location and dispersion of quantitative variables are presented as mean value and standard deviation for normally distributed variables and as median and interquartile range (1st-3rd quartile) for nonnormally distributed variables. Variables were tested for normal distribution using Kolmogorov-Smirnov and Shapiro-Wilk test.

A *p* value of ≤0.05 (5%) was considered statistically significant, and a value of ≤0.001 (0.1%) was deemed highly significant. In cross-sectional analysis, mean values of normally distributed variables were compared using the *T*-test for independent samples. We used the Mann–Whitney *U*-test for ordinally scaled variables or distribution skewness. For categorically scaled variables, Pearson's chi-square test was used. We used the Kruskal-Wallis H-test for comparisons involving more than two independent samples.

## 3. Results

The total group consisted of 903 children and adolescents who underwent at least one OGTT during their consultation at the obesity outpatient clinic, demonstrating a balanced gender distribution. The median age (1st-3rd quartile) at the initial OGTT was 14.2 (12.5-15.6) years. The median BMI was 32.7 (29.6-37.2) kg/m^2^, and the mean (±SD) BMI-SDS was 2.70 (±0.54). Further clinical characteristics of the patients are shown in Table 1.

**Figure 1:**
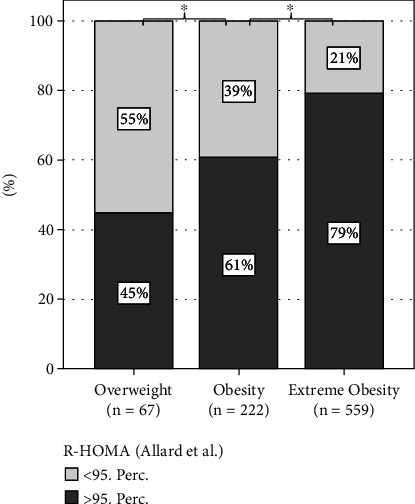
Prevalence of insulin resistance in children with overweight or obesity according to weight classification. ^∗^*p* < 0.05; *n*: number; perc: percentile.

Impaired glucose regulation, which includes impaired glucose tolerance (IGT), impaired fasting glycemia (IFG), and type 2 diabetes mellitus (T2D), was present in 12.7% of all patients. 9.4% of all patients presented with IGT, 1.2% with T2D, and 4.5% with IFG. 1.7% of all patients showed an overlapping between IFG and IGT, and 0.8% were diagnosed with both IFG and T2D. Insulin resistance was observed in 72.3% of the cohort based on the percentiles defined by Allard et al. [24].  Figure 1 illustrates a direct correlation, showing an increasing proportion of patients with insulin resistance corresponding to higher weight categories.

In a pilot study, we conducted genetic testing on twelve patients with suspected insulin secretion disorder.  Table 2 shows the results of targeted enrichment and provides clinical characteristics of patients with genetic variants. In 5 out of 12 patients, heterozygous mutations of known diabetes genes or genes associated with pancreas development were detected.

We found mutations in the ABCC8 gene in three patients; all of them showed an insulin secretion disorder during their OGTT. These individuals were diagnosed around the age of 12. One patient met the criteria for T2D when performing the genetic diagnostics. In the further course, it was possible to stabilize the metabolic situation in 2 out of 3 patients with ABCC8 mutation through diet and physical activity. One patient received metformin and had to switch to insulin therapy (ICT) later.

Additionally, a GCK gene mutation was identified in one patient diagnosed with IGT. This patient's father is also known to have a diabetic condition that is adequately managed by diet. In a 17-year-old female patient with T2D, mutations in two candidate genes (PTF1A and GLI2) were identified. This patient also has a positive family history. She was successfully treated with oral antidiabetic drugs (metformin).

**Figure 2:**
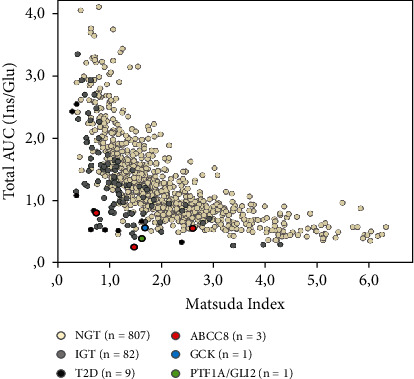
Hyperbolic relationship of insulin secretion (measured by Matsuda index) and insulin sensitivity (measured by Total AUC (Ins/Glu)). NGT: normal glucose tolerance; IGT: impaired glucose tolerance; T2D: type 2 diabetes; Ins: insulin; Glu: glucose; AUC: area under the curve; ABCC8: ATP binding cassette subfamily C member 8; GCK: glucokinase; PTF1A: pancreas associated transcription factor 1a, GLI2: GLI family zinc finger 2.

Figure 2 illustrates insulin sensitivity (Matsuda index) as a function of insulin secretion (Total AUC (Ins/Glu)); the product of both is also known as the disposition index (ISSI-2I). These parameters exhibit a hyperbolic correlation. Among children and adolescents with normal glucose tolerance, increased insulin resistance corresponds to heightened insulin secretion. The median disposition index in this group was 2.07 (1.74-2.50).

However, in patients with IGT and T2D, increased insulin resistance is not adequately balanced by increased insulin secretion, indicating a relative insulin deficiency. ISSI-2 decreased significantly (*p* < 0.001) in these groups and was only 1.30 (1.00-1.66) in the IGT group; patients with T2D showed a median ISSI-2 of 0.60 (0.44-0.86).

Additionally, subjects 1-5 from the genetic testing are highlighted in this figure. It becomes evident that, compared to the total cohort, these patients show a lower Matsuda index as a sign of impaired insulin sensitivity. However, the decreased beta-cell function is even more striking, shown on the *y*-axis by the Total AUC (Ins/Glu). Increased insulin secretion does not compensate for decreased insulin sensitivity in these patients, leading to a relative insulin deficiency. As a result, the hyperbolic relationship between the two parameters is lost. Consequently, these subjects are located below the cohort curve of patients with normal glucose tolerance.

## 4. Discussion

We screened a large pediatric cohort of children and adolescents with obesity by OGTT and identified in the second step a relevant percentage of patients with insulin secretion disorder and monogenic forms of diabetes.

In our findings, 4.5% of all patients showed elevated fasting glucose levels, 9.4% displayed impaired glucose tolerance, and 1.2% met the ADA criteria for T2D. Overall, we found IGR in 12.7% of the whole group. Similar to our cohort, in a very large cross-center study with >11,000 children and adolescents with obesity, an IGR was found in almost 13%, but the proportion of subjects with an IFG was slightly higher (6.0%), and only 5.5% showed an IGT [28]. Our study population comes from a high-risk group with most patients presenting with extreme obesity. As shown in Figure 1, many of the individuals present with an insulin resistance and the associated presence of IGT.

Comparatively, other studies have reported significantly higher rates of IGT (22.3-36.6%) and T2D (2.4-5.9%) [29, 30]. However, the mean BMI of study participants in both studies was higher, demonstrating the influence of even small weight differences. Divergent population profiles of the studies are another influencing factor. For example, people with African and Hispanic ethnicity, who make up a much larger proportion of the total population in the USA than in Germany, show much higher prevalence rates of IGR [31]. For Europe, ethnic risk groups have not yet been clearly defined, and prevalence and incidence rates vary significantly between European countries [32]. Overall, people of white ethnicity are considerably less likely to be affected by early T2D, which was also shown in the ethnic profile of our study.

Based on our cohort's findings and considering a prevalence of obesity of around 6.5%, approximately 6,000 children should be affected by T2D in Germany, Austria, and Switzerland. In the databases for children with obesity (APV) and children with diabetes (DPV), however, only about 1,100 cases were registered in 2010, which suggests a high number of unreported T2D cases [33]. From 2011 to 2019, the incidence of youth-onset type 2 diabetes in Germany (based on the APV registry) increased by 6.8% annually [34]. Targeted screening by OGTT in the high-risk group of children with obesity or extreme obesity and positive family history is therefore strongly indicated [35].

We calculated an oral disposition index (ISSI-2) using the Matsuda index as a surrogate parameter for insulin sensitivity and the AUC (Ins/Glu) to access beta-cell function. In our cohort, these two indices showed the expected hyperbolic relationship. The oral disposition index, a product of both parameters, notably decreased as glucose tolerance deteriorated, indicating a lack of beta-cell compensatory capacity in patients with IGT and T2D. Unfortunately, there is limited data on oral disposition indices in pediatric populations; therefore, comparing absolute values is impossible. Establishing reference values and percentiles in a large pediatric cohort would be helpful and necessary. Nevertheless, the disposition index is a good predictor for the development of IGT over two years [36]. This is supported by a study tracking over 1,500 nondiabetic patients over 7-8 years, where the disposition index emerged as the best predictor for T2D onset [37]. Furthermore, individuals at increased risk for T2D, such as first-degree relatives with a positive family history of T2D or women with PCOS, show a decreased disposition index even before diabetes manifestation, highlighting its diagnostic relevance as a screening parameter [17]. Recently, research on youth-onset type 2 diabetes has also shown the oral disposition index to be a reliable predictor of early glycemic control loss, highlighting its potential as an important prognostic tool in clinical assessments [38].

In the total cohort, 39 children and adolescents showed an IGR and suspected beta-cell dysfunction at the same time (insulin maximum in OGTT <200 mU/l). In a retrospective analysis of pediatric MODY cases with beta-cell dysfunction at our outpatient clinic, all patients showed low insulin levels under 200 mU/l (data not shown). We chose this cut-off because the secretory capacity of beta-cells plays a crucial role in childhood and adolescence. Even before the onset of T2D, children and adolescents show reduced insulin secretion [39]. As the disease progresses, the secretory capacity of beta-cells appears to decline much more rapidly than in adult comparison groups [40]. There also seems to be a synergistic effect between declining beta-cell function and insulin resistance in adolescents, accelerating this progression [32]. Unlike existing insulin resistance, beta-cell function appears to be a parameter with less variability [41], making it a potentially reliable screening parameter.

In our pilot study, we screened 12 out of 39 patients for rare pathogenic mutations of insulin secretion by targeted enrichment of multiple genomic regions. In 5 out of 12 patients, monogenic defects could be assigned (41.7%). Scaling this finding to the broader cohort of children and adolescents with suspected insulin secretion disorder suggests that about 16 subjects in this group might be affected. When considering the total cohort, this indicates a proportion of at least 16 out of 903 patients (1.7%) with monogenic forms of diabetes.

To our knowledge, no study with a comparable cohort that tested for monogenic forms of diabetes has been published. In the TODAY cohort with an ethnically heterogeneous profile, 488 children and adolescents with obesity and T2D were screened for monogenic forms of diabetes. Positive results were found in 4.5% of the total cohort, with pathogenic mutations mainly affecting the classic MODY genes GCK, HNF4A, and HNF1A [42]. In another study, patients diagnosed with early type 2 diabetes were examined for mutations in the HNF1A, HNF4A, and GCK genes; a monogenetic form of diabetes was diagnosed in 4% [43].

Our study has shown that genetic testing is useful even at the stage of IGT with the suspected presence of an insulin secretion disorder, as a notable proportion of children and adolescents were found to carry likely pathogenic mutations. The clinical presentation of MODY patients can vary significantly, and with an increasing prevalence of obesity and early T2D in childhood and adolescence, the accurate diagnosis of the correct form of diabetes becomes increasingly challenging [43]. While MODY patients were described as nonobese until a few years ago, more recent studies have shown that a relevant proportion of patients with monogenic diabetes are significantly overweight or obese [42, 44]. While T2D manifestation in normal-weight children and adolescents is almost excluded, the presence of obesity does not make it possible to clearly define T2D in this patient group [45, 46]. All genetically screened patients showed an insulin secretion disorder during the OGTT. However, in a cross-sectional study, it is impossible to differentiate between a monogenic form of diabetes and secondary beta-cell failure in patients with type 2 diabetes. As shown in the TODAY cohort, it is also possible to have monogenic diabetes and insulin resistance or T2D, making it impossible to distinguish based solely on clinical features [42]. A successful genetic diagnosis is essential for affected individuals regarding therapeutic consequences.

Genetic testing in our pilot cohort yielded five positive findings. On a pathophysiological level, the affected genes are all responsible for insulin secretion (GCK, ABCC8) or pancreas development (PTF1A, GLI2). Heterozygous ABCC8 mutations were found in three patients with obesity-associated insulin secretion disorder. These patients presented heterogeneously in terms of BMI, clinical course, and response to therapy. This variability is also shown in other studies, indicating that not only the functionality of the resulting mutant proteins but also allele expression, protective genetic mechanisms, and environmental factors have a crucial impact on clinical presentation [47]. Timmers et al. reviewed 55 cases with ABCC8 mutation (MODY 12) from literature. They found a large heterogeneity with various phenotypes ranging from mild IGT to insulin-dependent diabetes with sequelae [48]. A successful molecular genetic diagnosis has immense therapeutic significance for patients with ABCC8 mutations. If dietary therapy fails, the use of sulfonylureas is an excellent example of personalized medicine. These drugs bind to the receptor SUR1 (encoded by ABCC8) of the ATP-dependent potassium channel, closing it and stimulating insulin secretion [49]. As an analysis of the Diabetes Prospective Follow-Up (DPV) registry has shown, an early diagnosis is of great importance, as patients respond better to early sulfonylurea treatment, resulting in improved metabolic outcomes throughout the disease course [50].

In addition to the three patients with ABCC8 mutation, we identified one patient with a mutation in the GCK gene. GCK-MODY is one of the most common forms of MODY, occurring at a prevalence of 1 : 1000, with over 600 known GCK mutations documented [51]. Despite lifelong mildly elevated blood glucose levels, microvascular and macrovascular complications are rare in patients with GCK-MODY [52]. Although these patients are frequently treated with oral antidiabetics or insulin before genetic diagnosis, drug therapy is indicated in very few cases [53].

Patient number 5 showed two heterozygous mutations that might be related to the development of early-onset diabetes. The PTF1A gene encodes a transcription factor that plays a crucial role in pancreatic development [54]. Similar to ABCC8 mutations, the phenotype of these patients appears to be highly variable [55]. Additionally, the patient was found to have a likely pathogenic mutation in GLI2. This gene plays a role in adipogenesis and beta-cell differentiation and could thus explain the development of early-onset T2D in this patient [56, 57].

In 60% of the pilot group, we did not find any mutations in the candidate genes. As described before, some of these patients probably show a secondary beta-cell failure as part of their polygenic T2D. However, in future studies, it would be interesting and necessary to look deeper into the genetic data (exome or whole genome sequencing) to discover new genes and expand our understanding of beta-cell dysfunction.

### 4.1. Limitations

Our study has some limitations. Beta-cell function and insulin resistance were assessed based on indices calculated from glucose and insulin values obtained during the OGTT. In different studies, the OGTT as a diagnostic tool shows high variability and partly limited reproducibility [58, 59]. The gold standard for the determination of beta-cell function and insulin resistance would be the hyperinsulinemic-euglycemic clamp technique, which is not feasible in clinical routine [12]. Nonetheless, the calculated indices show good correlations, justifying their use as surrogate parameters [60]. For instance, we computed the oral disposition index (ISSI-2) by combining the Matsuda index and Total AUC (Ins/Glu), aiming to capture both pathophysiological cornerstones in the development of T2D. Studies have confirmed good correlations of this index with the actual disposition index [27].

The genetic study included only a pilot cohort of 12 patients. Since there are no validated reference values or percentiles for the course of insulin levels during OGTT, we chose a nonvalidated insulin cut-off of 200 mU/l to define suspected insulin secretion disorders in this initial study. Thus, a group of patients with reasonable suspicion of a genetically determined insulin secretion disorder was defined. In the context of a larger study, the examination of patients with abnormal indices of insulin secretion (Total AUC (Ins/Glu), ISSI-2) would prove highly beneficial. In addition, genetic diagnosis and comparing patients with extreme insulin resistance would be equally interesting and necessary.

This study is solely focused on genetic diagnostics in an obese study population. It would be interesting to examine a nonobese comparison cohort for the presence of monogenetic mutations in a subsequent study. Furthermore, the focus of this study was primarily on different indices as surrogate parameters of beta-cell function and insulin resistance. In routine clinical practice, family history, consanguinity, age at manifestation, and the determination of autoantibodies are also important for the diagnosis and identification of possible monogenetic forms of diabetes.

## 5. Conclusion

In our study involving a large cohort of obese children and adolescents, a significant proportion displayed impaired glucose regulation within the context of metabolic syndrome. Performing an OGTT provides the opportunity to calculate an oral disposition index. A low disposition index may indicate either beta-cell decompensation in the face of insulin resistance or the presence of a genetic disorder affecting insulin secretion, potentially involving variants in monogenic diabetes genes. A successful molecular genetic diagnosis is crucial for affected individuals and their families, as it is possible to enable individualized diabetes therapy and identify at-risk family members.

## Figures and Tables

**Table 1:** Clinical characteristics of the study participants.

Parameter	Number (%)
*Sex*	
Male	*n* = 451 (49.9%)
Female	*n* = 452 (50.1%)
*Age (years)*	
Median (1-3 quartile)	14.2 (12.5-15.6)
(Range)	(2.0-19.1)
*BMI (kg/m^2^)*	
Median (1-3 quartile)	32.7 (29.6-37.2)
(Range)	(19.9-57.7)
*BMI-SDS*	
Mean value (±SD)	2.70 (±0.54)
(Range)	(0.69-4.30)
*Weight*	
Overweight	*n* = 67 (7.9%)
Obese	*n* = 221 (25.9%)
Extremely obese	*n* = 560 (65.7%)
*Tanner stage*	
I: prepubertal	*n* = 43 (7.2%)
II/III: early puberty	*n* = 171 (28.7%)
IV/V: late puberty	*n* = 381 (64.0%)
Missing	*n* = 308

BMI: body mass index; SDS: standard deviation score; SD: standard deviation; *n* number.

**Table 2:** Genetic findings and clinical characteristics of patients with MODY.

Subject	1	2	3	4	5
Sex	M	M	F	M	F
Ethnicity	Tunisian/Caucasian	Caucasian	Turkish	German	Serbo-Croatian
Glucose tolerance	IGT	IGT	T2D	IGT	T2D
Age (years)	12.9	12.2	11.3	15.1	16.8
BMI (SD-score)	40 (3.1)	26.8 (2.06)	28.1 (2.37)	28.7 (2.06)	33.2 (2.58)
Therapy	Metformin, insulin after 2 years	Diet	Diet	Diet	Metformin
Gene	ABCC8 heterozygous	ABCC8 heterozygous	ABCC8 heterozygous	GCK heterozygous	PTF1A and GLI2 heterozygous
DNA-exchange	c.1836G>T	c.1616A>G	c.1616A>G	c.626C>T	c.499G>A c.4145G>A
Amino acid exchange	p.Glu612Asp	p.Tyr539Cys	p.Tyr539Cys	p.Thr209Met	p.Ala167Thr p.Arg1382His
Disease value	Disease-causing	Disease-causing	Disease-causing	Disease-causing	Disease-causing

IGT: impaired glucose tolerance; T2D: type 2 diabetes; Ins: insulin; Glu: glucose; AUC: area under the curve; M: male; F: female; BMI: body mass index; SD: standard deviation; ABCC8: ATP binding cassette subfamily C member 8; GCK: glucokinase; PTF1A: pancreas associated transcription factor 1a; GLI2: GLI family zinc finger 2.

## Data Availability

The data supporting this study's findings are available upon reasonable request.

## Supplementary Materials

Supplementary Materials Table S1: calculation of indices—this table contains the formulas for calculating the indices used above (HOMA-IR, Matsuda index, Total AUC (Ins/Glu), oral disposition index (ISSI-2)). Table S2: genes of targeted enrichment—this table contains a list and description of all genes that were examined as part of genetic diagnostics (targeted enrichment).
