# Supplementary material for: Insulin Secretion Defect in Children and Adolescents with Obesity: Clinical and Molecular Genetic Characterization
Source: J Diabetes Res. 2024 Mar 20;2024:5558634. doi: 10.1155/2024/5558634 (PMC10977255; doi:10.1155/2024/5558634)
Supplement: Supplementary Materials — Table S1: calculation of indices—this table contains the formulas for calculating the indices used above (HOMA-IR, Matsuda index, Total AUC (Ins/Glu), oral disposition index (ISSI-2)). Table S2: genes of targeted enrichment—this table contains a list and description of all genes that were examined as part of genetic diagnostics (targeted enrichment). [file 5558634.f1.docx]

**Supplementary materials**

Table S1: Calculation of Indices

| **Index** | **Calculation** | **Surrogate Parameter of** |
| --- | --- | --- |
| **HOMA-IR** | $\frac{I_{0} \left( \frac{\mu U}{ml} \right)\times G_{0} \left( \frac{mg}{dl} \right)}{405}$ | Hepatic insulin resistance |
| **WBISI**  **(Matsuda Index)** | $\frac{10,000}{\sqrt{\begin{aligned} I_{0}\left( \frac{mU}{l} \right)\times G_{0}\left( \frac{mg}{dl} \right)\times\\ I_{Mean value} \times G_{Mean value} \end{aligned}}}$ | Peripheral insulin sensitivity |
| **Total AUC _Ins/Glu_** | $\frac{I_{0}+2\times\left( I_{30}+I_{60}+I_{90} \right)+I_{120}}{G_{0}+2\times\left( G_{30}+G_{60}+G_{90} \right)+G_{120}}$ | Betacell function |
| **ISSI-2** | Total AUC _Ins/Glu_ x WBISI | Disposition Index |

HOMA-IR: Homeostatic Model Assessment for Insulin Resistance; WBISI: Matsuda-Index; AUC: Area under the Curve; Ins: Insulin; Glu: Glucose; ISSI-2: insulin secretion-sensitivity index-2/oral Disposition Index

Table S2: Genes of Targeted Enrichment

| **Target Region (Gene)** | **Up-stream Bases** | **Down-stream Bases** | **Chro-mo-some** | **Start** | **Stop** | **Selec-ted Tar-gets** | **Total Targets** | **Target Type** | **Cove-rage** | **Criteria to select target** |
| --- | --- | --- | --- | --- | --- | --- | --- | --- | --- | --- |
| PROX1 | 100 | 100 | 1 | 214161178 | 214214947 | 6 | 6 | Exon | 100 | T2D susceptibility |
| Nfasc | 100 | 100 | 1 | 204797682 | 204992050 | 34 | 34 | Exon | 100 | diabetes, experimental |
| SLC19A2 | 100 | 100 | 1 | 169433049 | 169455308 | 6 | 6 | Exon | 99 | syndromic diabetes |
| LMNA | 100 | 100 | 1 | 156052269 | 156109980 | 21 | 21 | Exon | 100 | T2D susceptibility |
| NOTCH2 | 100 | 100 | 1 | 120454076 | 120612417 | 35 | 35 | Exon | 99 | T2D susceptibility |
| SLC16A1 | 100 | 100 | 1 | 113454370 | 113499075 | 6 | 6 | Exon | 99 | congenital hyperinsulinism (CHI) |
| NR0B2 | 100 | 100 | 1 | 27237875 | 27240667 | 2 | 2 | Exon | 96 | T2D susceptibility |
| LDLRAD2 | 100 | 100 | 1 | 22138658 | 22151814 | 5 | 5 | Exon | 99 | T2D susceptibility |
| HDAC4 | 100 | 100 | 2 | 239969764 | 240322743 | 27 | 27 | Exon | 99 | monogenic, MODY-like diabetes |
| IRS1 | 100 | 100 | 2 | 227595933 | 227663606 | 2 | 2 | Exon | 99 | T2D susceptibility |
| IGFBP2 | 100 | 100 | 2 | 217498027 | 217529258 | 4 | 4 | Exon | 100 | T2D susceptibility |
| NEUROD1 | 100 | 100 | 2 | 182540733 | 182545492 | 2 | 2 | Exon | 100 | syndromic diabetes,  T2D susceptibility |
| GPD2 | 100 | 100 | 2 | 157291865 | 157443015 | 18 | 18 | Exon | 99 | T2D susceptibility |
| RND3 | 100 | 100 | 2 | 151324607 | 151344309 | 8 | 8 | Exon | 100 | T2D susceptibility |
| GLI2 | 100 | 100 | 2 | 121554767 | 121750329 | 13 | 13 | Exon | 100 | diabetes, experimental, T2D susceptibility |
| EIF2AK3 | 100 | 100 | 2 | 88856159 | 88927194 | 17 | 17 | Exon | 99 | syndromic diabetes |
| BCL11A | 0 | 0 | 2 | 60678302 | 60780633 | 7 | 7 | Exon | 99 | T2D susceptibility |
| GCKR | 100 | 100 | 2 | 27719606 | 27746650 | 19 | 19 | Exon | 100 | T2D susceptibility |
| KLF11 | 100 | 100 | 2 | 10183582 | 10195063 | 6 | 6 | Exon | 100 | T2D susceptibility |
| SLC2A2 | 100 | 100 | 3 | 170714037 | 170744868 | 11 | 11 | Exon | 100 | T2D susceptibility |
| ADCY5 | 0 | 0 | 3 | 123001143 | 123167392 | 22 | 22 | Exon | 100 | T2D susceptibility |
| SLMAP | 100 | 100 | 3 | 57743074 | 57914994 | 21 | 21 | Exon | 100 | T2D susceptibility |
| APPL1 | 100 | 100 | 3 | 57261665 | 57307598 | 22 | 22 | Exon | 100 | monogenic, MODY14 |
| UBE2E2 | 100 | 100 | 3 | 23244684 | 23632396 | 6 | 6 | Exon | 100 | T2D susceptibility |
| NR2C2 | 50 | 50 | 3 | 14989186 | 15090830 | 15 | 15 | Exon | 100 | T2D susceptibility |
| PPARG | 100 | 100 | 3 | 12329249 | 12475955 | 10 | 10 | Exon | 98 | T2D susceptibility |
| HADH | 100 | 100 | 4 | 108910770 | 108956431 | 9 | 9 | Exon | 100 | congenital hyperinsulinism (CHI) |
| TRMT10A | 100 | 100 | 4 | 100467764 | 100485289 | 10 | 10 | Exon | 100 | syndromic diabetes |
| SPP1 | 100 | 100 | 4 | 88896702 | 88904663 | 8 | 8 | Exon | 97 | T2D susceptibility |
| WFS1 | 100 | 100 | 4 | 6271477 | 6305092 | 9 | 9 | Exon | 100 | syndromic diabetes, t2d susceptibility |
| SH3BP2 | 100 | 100 | 4 | 2794650 | 2842923 | 16 | 16 | Exon | 99 | diabetes, experimental |
| PIK3R1 | 100 | 100 | 5 | 67511484 | 67597749 | 19 | 19 | Exon | 100 | syndromic (lipodystrophy), T2D susceptibility |
| ISL1 | 100 | 100 | 5 | 50678858 | 50690663 | 6 | 6 | Exon | 100 | diabetes, experimental |
| PLAGL1 | 100 | 100 | 6 | 144261337 | 144385835 | 13 | 13 | Exon | 100 | monogenic, TNDM,  T2D susceptibility |
| ENPP1 | 100 | 100 | 6 | 132129056 | 132216395 | 25 | 25 | Exon | 100 | t2D susceptibility |
| RFX6 | 100 | 100 | 6 | 117198276 | 117253426 | 19 | 19 | Exon | 100 | syndromic diabetes, MODY-like |
| HMGA1 | 100 | 100 | 6 | 34204477 | 34214108 | 9 | 9 | Exon | 100 | T2D susceptibility |
| CDKAL1 | 100 | 100 | 6 | 20534588 | 21232734 | 16 | 16 | Exon | 100 | diabetes, experimental |
| MNX1 | 100 | 100 | 7 | 156797447 | 156803447 | 4 | 4 | Exon | 99 | syndromic diabetes |
| CPA1 | 100 | 100 | 7 | 130020190 | 130028049 | 10 | 10 | Exon | 100 | exocrine pancreas insufficiency |
| PAX4 | 100 | 100 | 7 | 127250246 | 127255880 | 9 | 9 | Exon | 99 | monogenic, MODY9 |
| GCK | 100 | 100 | 7 | 44183770 | 44229122 | 12 | 12 | Exon | 100 | monogenic, MODY2,  T2D susceptibility |
| SLC30A8 | 100 | 100 | 8 | 117962412 | 118189053 | 16 | 16 | Exon | 99 | T2D susceptibility |
| TP53INP1 | 100 | 100 | 8 | 95938100 | 95961715 | 5 | 5 | Exon | 100 | T2D susceptibility |
| SOX17 | 50 | 50 | 8 | 55370445 | 55373506 | 2 | 2 | Exon | 100 | diabetes, experimental |
| FGFR1 | 50 | 50 | 8 | 38268606 | 38326402 | 23 | 23 | Exon | 100 | diabetes, experimental |
| GATA4 | 100 | 100 | 8 | 11561617 | 11617609 | 7 | 7 | Exon | 100 | syndromic diabetes |
| BLK | 100 | 100 | 8 | 11351421 | 11422208 | 13 | 13 | Exon | 100 | monogenic, MODY11 |
| AGPAT2 | 100 | 100 | 9 | 139567495 | 139582011 | 6 | 6 | Exon | 100 | syndromic diabetes, lipodystrophy |
| CEL | 100 | 100 | 9 | 135937265 | 135947350 | 11 | 11 | Exon | 99 | monogenic, MODY8 |
| TLE1 | 100 | 100 | 9 | 84198498 | 84303696 | 20 | 20 | Exon | 99 | T2D susceptibility |
| TLE4 | 100 | 100 | 9 | 82186778 | 82341756 | 20 | 20 | Exon | 100 | T2D susceptibility |
| CDKN2B | 100 | 100 | 9 | 22002802 | 22009412 | 3 | 3 | Exon | 100 | T2D susceptibility |
| CDKN2A | 100 | 100 | 9 | 21967651 | 21994590 | 6 | 6 | Exon | 99 | T2D susceptibility |
| GLIS3 | 0 | 0 | 9 | 3824128 | 4300035 | 12 | 12 | Exon | 100 | syndromic diabetes |
| DOCK1 | 100 | 100 | 10 | 128593923 | 129250880 | 52 | 52 | Exon | 100 | diabetes, experimental |
| TCF7L2 | 100 | 100 | 10 | 114709909 | 114927536 | 20 | 20 | Exon | 100 | T2D susceptibility |
| GLUD1 | 100 | 100 | 10 | 88809859 | 88854876 | 13 | 13 | Exon | 100 | congenital hyperinsulinism (CHI) |
| SLC29A3 | 100 | 100 | 10 | 73078910 | 73123247 | 7 | 7 | Exon | 100 | syndromic diabetes |
| PCBD1 | 100 | 100 | 10 | 72643167 | 72648641 | 4 | 4 | Exon | 100 | monogenic, recessive |
| NEUROG3 | 100 | 100 | 10 | 71331691 | 71333310 | 2 | 2 | Exon | 100 | syndromic diabetes,  T2D susceptibility |
| SIRT1 | 100 | 100 | 10 | 69644327 | 69678247 | 10 | 10 | Exon | 100 | monogenic, autoimmune |
| PTF1A enhancer | 0 | 0 | 10 | 23502316 | 23510131 | 10 | 10 | Full  Region | 100 | syndromic diabetes, pancreas aplasia |
| PTF1A | 100 | 100 | 10 | 23481360 | 23483281 | 2 | 2 | Exon | 100 | syndromic diabetes,  T2D susceptibility |
| MTNR1B | 100 | 100 | 11 | 92702689 | 92716048 | 2 | 2 | Exon | 100 | T2D susceptibility |
| UCP2 | 100 | 100 | 11 | 73685616 | 73693989 | 8 | 8 | Exon | 99 | T2D susceptibility |
| BSCL2 | 100 | 100 | 11 | 62457634 | 62477191 | 15 | 15 | Exon | 100 | syndromic diabetes, lipodystrophy |
| PTPRJ | 100 | 100 | 11 | 48002010 | 48192494 | 26 | 26 | Exon | 99 | diabetes, experimental |
| PAX6 | 100 | 100 | 11 | 31806240 | 31839609 | 18 | 18 | Exon | 100 | syndromic diabetes (recessive) |
| ABCC8 | 100 | 100 | 11 | 17414332 | 17498549 | 39 | 39 | Exon | 100 | monogenic, NDM, MODY12, T2D susceptibility, CHI |
| KCNJ11 | 100 | 100 | 11 | 17406696 | 17410978 | 3 | 3 | Exon | 100 | monogenic, NDM, MODY13, T2D susceptibility, CHI |
| KCNQ1 | 100 | 100 | 11 | 2466121 | 2870440 | 17 | 17 | Exon | 100 | T2D susceptibility |
| INS | 100 | 100 | 11 | 2180909 | 2182539 | 5 | 5 | Exon | 100 | monogenic, NDM, MODY10,  T2D susceptibility |
| HNF1A | 100 | 100 | 12 | 121416449 | 121440414 | 10 | 10 | Exon | 100 | monogenic, MODY3 |
| NR2C1 | 50 | 50 | 12 | 95413955 | 95467454 | 16 | 16 | Exon | 100 | T2D susceptibility |
| CAPS2 | 100 | 100 | 12 | 75669659 | 75723936 | 18 | 18 | Exon | 100 | diabetes, experimental |
| GLI1 | 100 | 100 | 12 | 57853818 | 57866147 | 14 | 14 | Exon | 100 | diabetes, experimental |
| PHB2 | 100 | 100 | 12 | 7074415 | 7080016 | 10 | 10 | Exon | 100 | diabetes, experimental |
| MCF2L | 100 | 100 | 13 | 113623435 | 113754153 | 31 | 31 | Exon | 100 | T2D susceptibility |
| IRS2 | 100 | 100 | 13 | 110406084 | 110439014 | 2 | 2 | Exon | 100 | T2D susceptibility |
| PDX1 | 100 | 100 | 13 | 28494068 | 28500551 | 2 | 2 | Exon | 100 | monogenic, MODY4 |
| SPTB | 100 | 100 | 14 | 65212901 | 65289966 | 36 | 36 | Exon | 99 | T2D susceptibility |
| ERO1L | 100 | 100 | 14 | 53108505 | 53162519 | 16 | 16 | Exon | 100 | diabetes, experimental |
| LIPC | 100 | 100 | 15 | 58724075 | 58861173 | 9 | 9 | Exon | 100 | T2D susceptibility |
| GCGR | 100 | 100 | 17 | 79761910 | 79771989 | 14 | 14 | Exon | 100 | T2D susceptibility |
| SOX9 | 100 | 100 | 17 | 70117061 | 70122660 | 3 | 3 | Exon | 99 | diabetes, experimental |
| HNF1B | 100 | 100 | 17 | 36046334 | 36105196 | 10 | 10 | Exon | 100 | monogenic, MODY5 |
| SLC2A4 | 100 | 100 | 17 | 7184954 | 7191467 | 11 | 11 | Exon | 100 | T2D susceptibility |
| RAP1GAP2 | 100 | 100 | 17 | 2699632 | 2941135 | 25 | 25 | Exon | 100 | T2D susceptibility |
| IER3IP1 | 100 | 100 | 18 | 44681290 | 44702845 | 3 | 3 | Exon | 100 | syndromic diabetes, NDM |
| GATA6 | 100 | 100 | 18 | 19749304 | 19782591 | 7 | 7 | Exon | 100 | syndromic diabetes |
| SYT5 | 100 | 100 | 19 | 55684369 | 55691820 | 9 | 9 | Exon | 99 | T2D susceptibility |
| AKT2 | 100 | 100 | 19 | 40736124 | 40791402 | 15 | 15 | Exon | 100 | T2D susceptibility |
| RETN | 100 | 100 | 19 | 7733872 | 7735440 | 5 | 5 | Exon | 100 | T2D susceptibility |
| INSR | 100 | 100 | 19 | 7112166 | 7294111 | 22 | 22 | Exon | 100 | syndromic diabetes, T2D susceptibility |
| PTPN1 | 100 | 100 | 20 | 49126791 | 49201186 | 10 | 10 | Exon | 100 | T2D susceptibility |
| HNF4A | 100 | 100 | 20 | 42984341 | 43061585 | 14 | 14 | Exon | 100 | monogenic, MODY1 |
| TGIF2 | 50 | 50 | 20 | 35201826 | 35222405 | 6 | 6 | Exon | 100 | diabetes, experimental |
| FOXA2 | 50 | 50 | 20 | 22561592 | 22566151 | 4 | 4 | Exon | 100 | T2D susceptibility |
| NKX2-2 | 100 | 100 | 20 | 21491560 | 21494764 | 2 | 2 | Exon | 99 | diabetes, experimental |
| INSM1 | 100 | 100 | 20 | 20348665 | 20351692 | 1 | 1 | Exon | 100 | syndromic diabetes |
| HDAC8 | 100 | 100 | X | 71549266 | 71793053 | 14 | 14 | Exon | 99 | diabetes, experimental |
| FOXP3 | 100 | 100 | X | 49106797 | 49121388 | 12 | 12 | Exon | 100 | monogenic, autoimmune, x-linked |
